# Epidemiological and Genetic Characterization of Sapovirus in Patients with Acute Gastroenteritis in Valencia (Spain)

**DOI:** 10.3390/v13020184

**Published:** 2021-01-26

**Authors:** Sibele de Oliveira-Tozetto, Cristina Santiso-Bellón, Josep M. Ferrer-Chirivella, Noemi Navarro-Lleó, Susana Vila-Vicent, Jesús Rodríguez-Díaz, Javier Buesa

**Affiliations:** 1 Department of Microbiology, School of Medicine, University of Valencia, 46010 Valencia, Spain; sibele.tozetto@gmail.com (S.d.O.-T.); n0e_95@hotmail.com (N.N.-L.); Susana.Vila@uv.es (S.V.-V.); jesus.rodriguez@uv.es (J.R.-D.); 2 Microbiology Service, INCLIVA Health Research Institute, Hospital Clínico Universitario de Valencia, 46010 Valencia, Spain; ferrer.jmf@gmail.com

**Keywords:** sapovirus, acute gastroenteritis, real-time multiplex PCR, genotypes

## Abstract

Sapovirus is a common cause of acute gastroenteritis in all age groups. Sapovirus infections are seldom investigated in Spain, and its epidemiology in the country is not well known. The use of molecular diagnostic procedures has allowed a more frequent detection of sapoviruses in patients with diarrhea. A total of 2545 stool samples from patients with acute gastroenteritis attended from June 2018 to February 2020 at the Clinic University Hospital in Valencia, Spain, were analyzed by reverse transcription (RT) and real-time multiplex PCR (RT-PCR) to investigate the etiology of enteric infections. Sapovirus was the second enteric virus detected with a positive rate of 8%, behind norovirus (12.2%) and ahead of rotavirus (7.1%), astrovirus (4.9%) and enteric adenoviruses (2.9%). Most sapovirus infections occurred in infants and young children under 3 years of age (74%) with the highest prevalence in autumn and early winter. Coinfections were found in 25% of the patients with sapovirus diarrhea, mainly with other enteric viruses. Genotyping demonstrated the circulation of seven different genotypes during the study period, with a predominance of genotypes GI.1, GI.2, and GII.1. Phylogenetic analysis showed that genogroup GII strains form a cluster separated from genogroup GI and GV, being genotype GV.1 strains related to genotype GI.1 and GI.2 strains.

## 1. Introduction

Sapoviruses cause acute gastroenteritis in patients of all ages, with the highest prevalence in children under 5 years old. The burden of sapovirus disease has often been neglected due to the lack of commercial diagnostic tests [1]. The implementation of diagnostic molecular assays in clinical laboratories has contributed to an increased detection of sapovirus infections in recent years [2,3]. Sapoviruses have been reported worldwide to cause outbreaks of acute gastroenteritis mainly in daycare centers and healthcare facilities throughout the year, and are associated with 1–17% of diarrhea sporadic cases, usually in the cold season and in younger children [1,4]. They can be transmitted from person-to-person and by contaminated water or foods [5]. The incubation period of sapovirus infections varies from less than 1 to 4 days and generally cause a mild disease consisting of diarrhea and vomiting, and sometimes coinfections [6,7,8].

Sapoviruses are classified in the *Caliciviridae* family within the order *Picornavirales* (genus *Sapovirus*, species Sapporo virus) [9,10]. The human *Sapovirus* genome is a 7.1–7.7 kb single-stranded, positive-sense RNA molecule with a 3′-end poly(A) tail, which encompasses two or three open reading frames (ORF1, ORF2 and ORF3), according to the genogroup. The ORF1 encodes non-structural proteins (NS1–NS7) and a capsid protein (VP1, 60 kDa) [11,12,13], ORF2 encodes a minor capsid structural protein (VP2), whose expression was detected in vitro in cells infected with a porcine sapovirus using a reverse genetic system [14] and the ORF3 encodes proteins of unknown function [15,16].

Sequence analysis of the viral capsid VP1 gene is widely used for the genetic classification of sapoviruses and for the designation of new genogroups and/or genotypes [17]. Human and animals sapoviruses are classified into 19 genogroups based on available VP1 sequences (GI–GXIX). GI, GII, GIV and GV genogroups are known to infect humans whereas the remaining genogroups have been detected in swine (GIII and GV–GXI), sea lions (GV), mink (GXII), dogs (GXIII), bats (GXIV, GXVI–GXIX) and rats (GXV). Human sapoviruses can be further classified into 18 genotypes, with GI and GII subdivided into 7 genotypes (GI.1 to GI.7) and 8 genotypes (GII.1 to GII.8), respectively. GIV contains a single genotype (GIV.1) and GV was subdivided into two genotypes for humans (GV.1 and GV.2), one genotype for pigs (GV.3) and one for sea lions (GV.4) [17,18,19].

Sapoviruses, as well as other enteric viruses, have been increasingly detected in cases of acute gastroenteritis, causing approximately 4% of acute gastroenteritis outbreaks in Europe [20,21,22] and are also emerging worldwide [18,23,24,25]. In Spain, some studies have detected the presence of sapoviruses in a significant number of fecal samples from patients with acute gastroenteritis, demonstrating a dynamic evolution of genogroups and genotypes [26,27,28,29,30].

To contribute to the understanding of the role of sapovirus as an etiologic agent of acute gastroenteritis, this study aimed to analyze the prevalence and genetic characteristics of human sapoviruses in patients with acute gastroenteritis in Valencia, Spain, from June 2018 to February 2020.

## 2. Materials and Methods

### 2.1. Specimen Collection and Microbiological Diagnosis

From June 2018 to February 2020 a total of 2545 fecal samples from outpatients with acute gastroenteritis, both children and adults, were collected for bacterial and viral enteropathogens prospective detection by real-time multiplex PCR. The BD Max enteric viral panel combined with the enteric bacterial panel, and the *C. difficile* toxin B panel (BD Max Cdiff) (Becton Dickinson, Sparks, MD, USA) when requested, were used at the Clinical Microbiology Laboratory of the Hospital Clínico Universitario of Valencia to analyze the stools. Positive samples for enteropathogenic bacteria were further cultivated in the appropriate bacteriological media, and those positive for enteric viruses were kept at −20 °C for viral strain characterization. The BD Max enteric viral panel detects norovirus genogroup I (GI) and GII, rotavirus type A, adenovirus type F 40/41, human astrovirus and sapovirus (genogroups I, II, IV, and V). The testing procedure was performed according to the manufacturer’s instructions and all samples were anonymized once the diagnosis was done. The present study was carried out in accordance with the Declaration of Helsinki and was approved by the Ethics Committee of the Hospital Clínico Universitario of Valencia (Approval No. F-CE-GEva-15, 26 May 2015).

### 2.2. Nucleic Acid Extraction and Reverse Transcription

One-hundred and eighty-six sapovirus-positive specimens were analyzed for further viral genomic characterization by reverse transcription (RT) and hemi-nested PCR and sequencing. Clarified 10% sapovirus-positive stool suspensions were prepared in phosphate-buffered saline and RNA was extracted using TRIzol^®^ reagent (Invitrogen, Thermo Fisher Scientific) [31]. The RT reaction was performed with random primers and the SuperScript^®^ III reverse transcriptase (Life Technologies, Thermo Fisher Scientific, Waltham, MA, USA).

### 2.3. Amplification of Sapovirus Partial Capsid Gene

Once the cDNA was obtained, it was first amplified by conventional PCR with primers SV-F13/F14 and SV-R13/R14 previously described [15], to generate amplicons of approximately 800 bp. The conditions for the thermal cycling were as follows: initial denaturation at 95 °C for 3 min was followed by 35 cycles of 95 °C for 30 s, 48 °C for 30 s, and 74 °C for 45 s. A final incubation at 74 °C for 5 min was performed for complete extension. All samples, positive and negative from the first PCR, were analyzed by a nested PCR with primers SV-F22 and SV-R2 under identical conditions, to amplify a region of approximately 420 bp in the capsid gene [15]. The sequences of the primers used are shown in Table 1. Amplicons obtained in the nested PCR were sequenced for phylogenetic analyses.

### 2.4. Sequencing and Phylogenetic Analysis

When needed, amplicons were purified using the NZYGelpure kit (Nzytech, Lisboa, Portugal). Sequencing was carried out by the ABI Prism system (Applied Biosystems, Waltham, MA, USA) with the Big Dye Terminator V3.1 cycle sequencing kit and the 48-capillary ABI 3730 DNA analyzer. The quality of the sequences was evaluated with BioEdit software v7.0.0 [33] and the characterization of the genogroup and the genotype of the sequenced viral capsid genes was carried out based on NCBI-BLAST data. The sapovirus sequences obtained in this study were deposited in GenBank under accession numbers MT580810, MT58081, MT580812–MT580814, MT580815–MT580861, MT580862–MT580882, MT580883–MT580890, MT580891–MT580896, for the partial capsid sequences.

Sequences were aligned and analyzed with software programs Clustal X 2.0 [34], GeneDoc 2.7 and MEGA7 (Molecular Evolutionary Genetics Analysis v7.0) [35]. Phylogenetic trees were built by the maximum-likelihood method (bootstrap of 1000 replicates), with the sequences from this study and reference sequences retrieved from GenBank. Genotypes were assigned based on BLAST analyses and clustering with reference strains.

### 2.5. Statistical Analyses

The Mann–Whitney U and chi-squared tests were used to assess the equality of medians for two observation samples. The level of statistical significance was established as α = 0.05. Statistical analyses were performed by using IBM SPSS1 version 18 program (SPSS Inc., Chicago, IL, USA).

## 3. Results

### 3.1. Detection of Sapovirus and Other Enteropathogenic Agents

**Figure 1:**
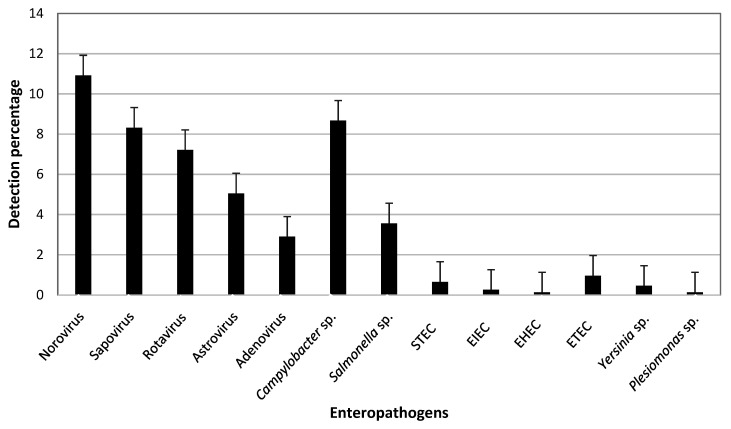
Microbiological findings in stool samples by real-time multiplex PCR (BD Max) from patients with acute gastroenteritis from June 2018 to February 2020 in Valencia (Spain). For simplicity, mixed infections are not shown. Abbreviations: STEC, Shiga toxin-producing *Escherichia coli*; EIEC, enteroinvasive *E. coli*; EHEC, enterohemorrhagic *E. coli*; ETEC, enterotoxigenic *E. coli*.

The BD Max enteric viral testing was performed in 2545 fecal samples rendering the following rates of virus detections: norovirus, 12.2%; sapovirus, 8%; rotavirus, 7.1%; astrovirus, 4.9%, and enteric adenovirus 2.9%. Among enteropathogenic bacteria, *Campylobacter* sp. (8.7%) and *Salmonella enterica* (3.6%) were the most frequently detected, followed by enterotoxigenic *E. coli* (ETEC) (1%), Shiga toxin-producing *E. coli* (STEC) (0.6%) and *Yersinia enterocolitica* (0.5%) (Figure 1). Amplicons were obtained in 88 (47.3%) of 186 sapovirus-positive samples analyzed by conventional PCR and hemi-nested PCR, and were then subjected to genetic characterization.

### 3.2. Temporal Distribution of Sapovirus

**Figure 2:**
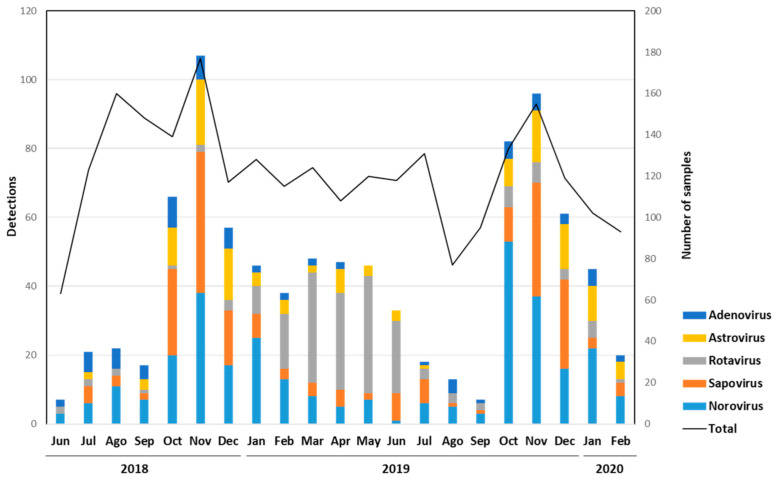
Enteric viruses detected in patients with acute gastroenteritis from June 2018 to February 2020 in Valencia, Spain.

**Figure 3:**
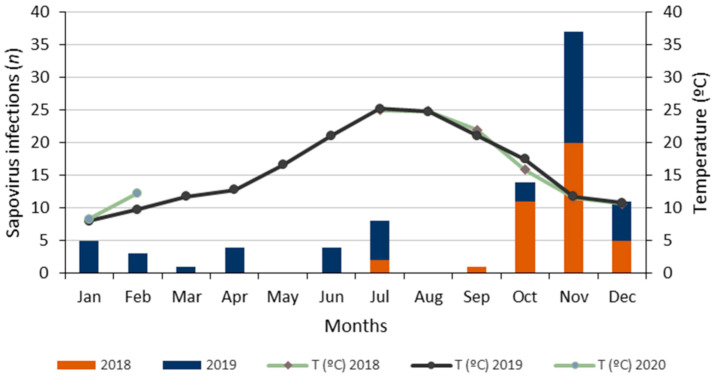
Cumulative numbers of sapovirus infections by months in Valencia (Spain) from June 2018 to February 2020. Monthly average temperatures (line).

The monthly distribution of sapovirus and other enteric virus infections was evaluated, detecting cases all year long, with a higher prevalence of sapovirus, norovirus and astrovirus in autumn and early winter, and a peak in November in both years 2018 and 2019. Rotavirus infections were more prevalent from February to June 2019 (Figure 2). The increase in the prevalence of sapovirus infections was coincidental with a drop in the average atmospheric temperatures (Figure 3).

### 3.3. Gender and Age Distribution of Sapovirus Infections

Of the 204 sapovirus strains detected, 55.9% were from male and 44.1% from female patients (Table 2). The age distribution of the cases indicates that most patients were infants and children under 2 years old (158/204; 77.4%). The greater impact on this age group is statistically significant (*p* < 0.05). Interestingly, only 5 cases (2.4%) were recorded in patients over 65 years old.

### 3.4. Coinfection with Other Enteropathogenic Agents

Coinfections with other enteropathogens were observed in 25% (51/204) of patients with sapovirus diarrhea (Table 3). Mixed infections were frequently detected with norovirus (27.4%), astrovirus (21.5%), and rotavirus (17.6%), but also occurred with adenovirus (7.8%), *Campylobacter jejuni* (9.8%), *Clostridioides difficile* (1.9%), *Salmonella enterica* (1.9%) and Shiga toxin-producing *Escherichia coli* (STEC) (1.9%). Coinfections of sapovirus and other two pathogens were also observed (norovirus and rotavirus, norovirus and adenovirus, norovirus and *S. enterica*, adenovirus and astrovirus). These coinfections were detected predominantly in infants, followed by the group of children aged 1–2 years.

### 3.5. Genetic Characterization

#### 3.5.1. Genogroups and Genotypes

**Figure 4:**
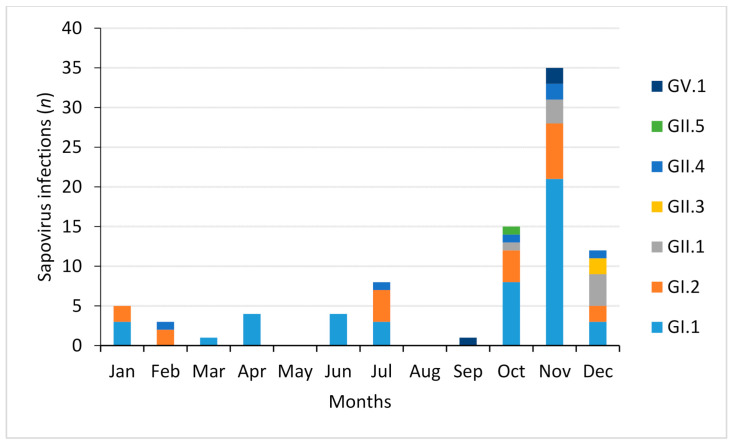
Sapovirus genotypes identified in patients with gastroenteritis from June 2018 to February 2020 in Valencia, Spain.

From a total of 88 positive samples with suitable sequences for genetic characterization, 68 corresponded to genogroup GI (77.3%), 17 (19.3%) to genogroup GII and 3 to genogroup GV (3.4%). In total 7 different sapovirus genotypes were identified from the 88 characterized strains. Genogroup I sequences were classified into GI.1 (47/68; 69.1%) (the predominant genotype) and GI.2 (21/68; 30.9%). On the other hand, genogroup II sequences have been divided into GII.1 (8/17; 47%), GII.3 (2/17; 11.8%), GII.4 (6/17; 35.3%) and GII.5 (1/17, 5.9%). The three genogroup GV strains were classified as genotype GV.1. The temporal distribution of the different genotypes showed a predominance of GI.1 and GI.2 during autumn and winter, in the months of October, November and December (Figure 4). However, some cases were also caused by these genotypes in summer. Genotype GII.1 was found in late autumn and early winter. GII.3 and GII.5 occurred only in December and October, respectively. On the other hand, genotype GII.4 was detected in autumn and winter (October, November, December and February) and July, at low percentages.

#### 3.5.2. Phylogenetic Analysis

**Figure 5:**
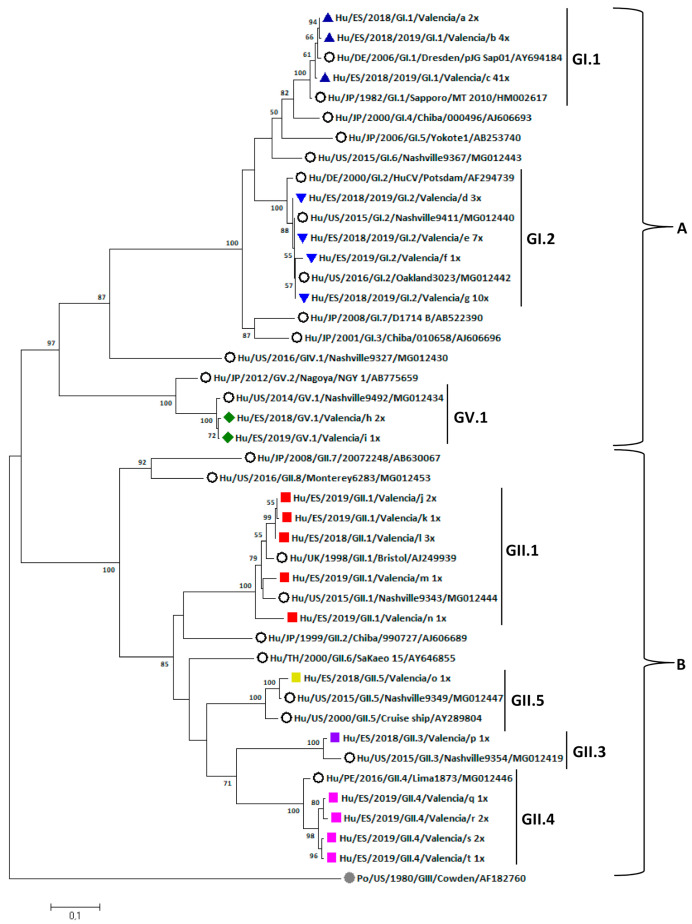
Phylogenetic analysis of the partial capsid gene of sapoviruses collected in Valencia, Spain from July 2018 to February 2020. Phylogenicity was inferred using the Maximum-Likelihood method based on the Kimura 2-parameter model with a bootstrap of 1000 replicates. Bootstrap values >50% are shown. The tree was condensed by removing sequences of the same genotype that clustered together. The tree is drawn to scale; the branch lengths measure the number of substitutions per site. The analyses included 111 sequences (87 samples and 24 references). References strains are represented by their GenBank accession numbers and indicated with empty circles. The outgroup is indicated with filled circle in grey. Sequences obtained in this study are indicated as follows: ▲ in dark blue, GI.1; ▼ in blue, GI.2; ■ in red, GII.1; ■ in purple, GII.3; ■ in pink, GII.4; ■ in yellow, GII.5; ■ in green, GV.1. The letters a-s represents the number of samples analyzed and their respective GenBank access numbers. a = MT580822 and 580837; b = MT580821, 580834, 580841, and 580854; c = MT580815-MT580820, MT580823-MT580833, MT580835-MT580836, MT580838-MT580840, MT580842-MT580853, and MT580855-MT580861; d = MT580862-MT580863 and MT580870; e = MT580865, MT580871, MT580877, and MT580879-MT580882; f = MT580872; g = MT580864, MT580866-MT580869, MT580873-MT580876, and MT580878; h = MT580812 and MT580813; i = MT580814; j = MT580888; k = MT580886 and MT580889; l = MT580883-MT580885; m = MT580887; n = MT580890; o = MT580811; p = MT580810; q = MT580891; r = MT580894 and MT580895; s = MT580892 and MT580893; t = MT580896.

Phylogenetic analyses of the partial VP1 gene of 88 sapovirus strains in this study and reference strains (NCBI-GenBank) demonstrates that they cluster into two main groups, one including genotypes GI.1, GI.2, GIV.1 and GV.1 (cluster A) and the other one including genotypes GII.1, GII.3, GII.4 and GII.5 (cluster B) (Figure 5). Within cluster A, GV.1 forms a subcluster independent from the rest of the group. The GV strains share a common ancestor with the reference strain Nashville9492 (MG012434). In addition, in GI genogroup, GI.1 and GI.2 form two separated subclusters. GI.1 strains share a common ancestor with the original reference strain Sapporo (1982) (HM002617) and are closer to the Dresden reference strain (2006) (AY694184). The GI.2 strains detected in this study are closer to the more recent reference strains Nashville9367 (MG012443) and Oakland3023 (MG012442), from 2015 and 2016, respectively, while the older reference strain Potsdam 2000 (AF294739) is more distant. Regarding genogroup GII, two subgroups were observed, one including reference strains for genotypes GII.7 and GII.8 (20072248-AB6330067 and Monterey6283-MG012453, respectively) and another one including all the GII strains from this study. The GII.1 strains share ancestors with an older reference strain (Bristol, 1998-AJ249939) and a newer 2015 strain (Nashville9343-MG012444). The GII.5 strain found in this study shares an ancestor with a more recent reference strain (Nashville9349-MG012447) and these are grouped with a 2000-year reference strain (Cruise ship-AY289804). The GII.3 genotype clusters with a 2015 reference strain (Nashville9354-MG012419). Finally, it appears that the subcluster formed by the GII.4 genotype strains, in which many strains of this study are grouped, form an independent group with respect to the reference strain Lima1873-MG012446.

## 4. Discussion

Molecular techniques for enteropathogen detection provide comprehensive, sensitive, and rapid alternatives for diagnosing infectious diarrhea, with clear advantages over conventional methods. It has been reported a negative percent agreement (NPA) of 99.4% for all viral targets in the BD Max enteric viral panel, with positive percent agreement (PPA) values for norovirus, astrovirus, rotavirus, and adenovirus of 92.8%, 93.0%, 100% and 95.6%, respectively [3]. Sapovirus had a PPA of 84.9% in those analyses, which was lower than that found in a previous study (PPA, 100%) involving the BioFire FilmArray GI panel (bioMérieux) [2].

In the present study it was found that noroviruses and sapoviruses were the most prevalent enteric viruses in the study population, with positive rates of 12.22% and 8.09%, respectively, followed by rotaviruses (7.11%), human astroviruses (4.91%) and enteric adenoviruses (2.9%). The detection frequency of sapovirus has been reported to range from 1 to 17% of diarrhea cases [1], although it varies across age groups and geographical locations. Similar results have been obtained in other studies performed in the post-rotavirus vaccine era, in which norovirus, sapovirus, and rotavirus infection were identified in this order of frequency [20,36,37,38]. Although many studies show that norovirus remains the most common enteric virus detected, sapovirus is increasingly being found. In a previous study carried out in 2010–2011 in Northwest Spain (Galicia) a higher sapovirus prevalence (15.6%) was reported [27]. By contrast, in a two-year prospective study of gastroenteritis outbreaks in Catalonia in 2010–2011, sapovirus was only identified in two outbreaks of person-to-person transmission in children, and in one outbreak of foodborne transmission with coinfection with norovirus [26]. In that study, in 98% of 101 outbreaks investigated the causative agent was norovirus.

Other enteric pathogens besides sapovirus were detected in 25% of the fecal samples in our study. Most of these co-detections involved other enteric viruses (norovirus, astrovirus, rotavirus and enteric adenovirus, in this order), but also bacteria (*Campylobacter jejuni*, *Salmonella enterica*, Shiga toxin-producing *E. coli* (STEC) and B toxin-producing *C. difficile*). Many other studies have reported mixed infections of sapovirus with other enteric pathogens [27,39,40]. Interestingly, the detection of sapovirus in healthy controls has also been reported [1,41]. However, the multinational Malnutrition and Enteric Disease (MAL-ED) cohort study estimated that sapovirus has the third highest attributable incidence for diarrhea among children under 1 year of age and the second-highest attributable incidence between 1 and 2 years of age, just behind *Shigella* sp. [42].

It is remarkable that in this study most sapovirus infections occurred in infants and young children under 2 years old (77.4%). Other authors report the same findings [20,27], although it has been also described an increase in sapovirus prevalence in elders above 60 years old [27], which was not confirmed in our study. In one survey conducted in Germany it was found that in patients older than 14 years, sapoviruses were only detected in immunocompromised individuals [43], which was not the case of our patients.

The highest number of sapovirus infections occurred in autumn and early winter, with a peak in November in both years 2018 and 2019. In a previous study conducted in our country in 2010–2011, the highest prevalence was found in September, October and late winter (February), which is not coincidental with our findings [27].

It has been reported that the most common human sapovirus genogroups worldwide are GI and GII [21,23]. Our results are coincidental, as 68 strains out of 88 corresponded to GI (77.3%) and 17 to GII (19.3%), and only 3 strains were GV (3.4%). In our study, seven different sapovirus genotypes were identified. GI.1 and GII.2 were the two most common, followed by GII.1, the same predominant genotypes found in 2010–2011 in Galicia (Spain) by Varela et al. [27]. It has been reported that these three dominant genotypes are more frequent not only in the first year of life but also in adults [1,27]. Other genotypes detected in our study were also found previously circulating in Galicia (GII.4, GII.5, GV.1); however, we have not found GI.3, GII.2 and GIV.1 genotypes, and they did not detect GII.3 strains. By phylogenetic analysis it was clearly demonstrated that genogroup GII strains form a cluster separated from genogroup GI and GV, being genotype GV.1 strains related to genotype GI.1 and GI.2 strains.

To trace the distribution of sapovirus genotypes in different settings and geographical areas is relevant, as this information is epidemiologically useful to design and develop broadly protective sapovirus vaccines [1]. Even though several sapovirus recombinant strains of both intragenogroup and intergenogroup recombinations have been reported [18,25,44], sequence analyses from five major genotypes (GI.1, GI.2, GII.1, GII.3, and GIV.1) have shown, contrary to noroviruses, limited intra-genotypic diversification for over 40 years [45]. The relationship between sapovirus genotypes and serotypes is still unknown and more studies are needed to clarify this question, but this observation gives hope to the future development of sapovirus vaccines.

## Figures and Tables

**Table 1:** Primers used in this study to detect and characterize sapovirus strains [15,32].

Use	Prime/Probe	Nucleotide Sequence (5′–3′)	Positions *
Capsid genogrouping	SV-F13	GAYYWGGCYCTCGCYACCTAC	5074–5094
	SV-F14	GAACAAGCTGTGGCATGCTAC	5074–5094
	SV-R13	GGTGANAYNCCATTKTCCAT	5876–5861
	SV-R14	GGTGAGMMYCCATTCTCCAT	5876–5861
	SV-F22	SMWAWTAGTGTTTGARATG	5154–5172
	SV-R2	GWGGGRTCAACMCCWGGTGG	5591–5572

* Positions in complete sapovirus GI genogroup sequence (Manchester, accession no. X86560) [32]. Codes for generate bases: Y = C, T; W = A, T; N = A, C, G, T; K = G, T; M = A, C; R = A, G.

**Table 2:** Gender and age of patients with sapovirus infection.

Age (Years)	N° Male	N° Female	Total (%)
0–2	89	69	158 (77.4)
3–5	8	14	22 (10.8)
6–12	7	2	9 (4.4)
13–18	2	0	2 (1)
19–59	5	3	8 (3.9)
>60	3	2	5 (2.4)
Total (%)	114 (55.9)	90 (44.1)	204 (100)

**Table 3:** Distribution of coinfections of sapovirus with other enteropathogens in patients with acute gastroenteritis in Valencia, Spain.

Coinfection with:	N° of Cases (%)
Norovirus	14 (27.4)
Astrovirus	11 (21.5)
Rotavirus	9 (17.6)
Enteric adenovirus	4 (7.8)
*Campylobacter jejuni*	5 (9.8)
*Clostridioides difficile*	1 (1.9)
*Salmonella enterica*	1 (1.9)
Shiga toxin-producing *E. coli*	1 (1.9)
Norovirus and rotavirus	2 (3.8)
Norovirus and adenovirus	1 (1.9)
Norovirus and *Salmonella* sp.	1 (1.9)
Adenovirus and astrovirus	1 (1.9)
Total (%)	51 (100)

## Data Availability

Sequences obtained in this study are available at the NCBI GeneBank repository with accession numbers MT580812-MT580896.
